# No Robust Association between Static Markers of Testosterone and Facets of Socio-Economic Decision Making

**DOI:** 10.3389/fnbeh.2017.00250

**Published:** 2017-12-20

**Authors:** Laura Kaltwasser, Una Mikac, Vesna Buško, Andrea Hildebrandt

**Affiliations:** ^1^Berlin School of Mind and Brain, Humboldt-Universität zu Berlin, Berlin, Germany; ^2^Faculty of Humanities and Social Sciences, University of Zagreb, Zagreb, Croatia; ^3^Department of Psychology, Ernst-Moritz-Arndt-Universität Greifswald, Greifswald, Germany

**Keywords:** testosterone, 2D:4D, facial width-to-height ratio, economic decision making, social preferences, assertiveness

## Abstract

Digit ratio (2D:4D) and facial width-to-height ratio (WHR) are supposedly static indicators of testosterone exposition during prenatal and pubertal lifetime, respectively. Both measures have been linked to aggressive and assertive behavior in laboratory economic games, as well as in real world scenarios. Most of the research—often limited to male subjects—considers the associations between these behaviors, traits, and hormonal markers separately for 2D:4D and WHR. Reported associations are weak and volatile. In the present study we had independent raters assess 2D:4D and WHR in a sample of *N* = 175 participants who played the ultimatum game (UG). Respondent behavior in UG captures the tendency to reject unfair offers (negative reciprocity). If unfair UG offers are seen as provocations, then individuals with stronger testosterone exposition may be more prone to reject such offers. Economists argue that negative reciprocity reflects altruistic punishment, since the rejecting individual is sacrificing own resources. However, recent studies suggest that self-interest, in terms of status defense plays a substantial role in decisions to reject unfair offers. We also assessed social preferences by social value orientation and assertiveness via self-report. By applying structural equation modeling we estimated the latent level association of 2D:4D and WHR with negative reciprocity, assertiveness and prosociality in both sexes. Results revealed no robust association between any of the trait measures and hormonal markers. The measures of 2D:4D and WHR were not related with each other. Multigroup models based on sex suggested invariance of factor loadings allowing to compare hormone-behavior relationships of females and males. Only when collapsing across sex greater WHR was weakly associated with assertiveness, suggesting that individuals with wider faces tend to express greater status defense. Only the right hand 2D:4D was weakly associated with prosocial behavior, indicating that individuals with lower prenatal testosterone exposure are more cooperative. Rejection behavior in UG was not related with 2D:4D nor WHR in any of the models. There were also no curvilinear associations between 2D:4D and prosociality as theorized in the literature. Our results suggest that previous studies over-estimated the role of static markers of testosterone in accounting for aggression and competition behavior in males.

## Introduction

### The impact of testosterone

The steroid hormone testosterone, produced in the male testes, and to a lesser extent in female ovaries, circulates the human brain throughout life and it is assumed to impact behavior and its development. Relationships between hormonal activity and behavior are complex, consisting of both endocrine effects on behavior and, vice versa, behavioral effects onto endocrine function. On the one hand, endocrines have been shown to affect attachment and sex (Carter, 1998; Insel and Young, 2001), aggression (Koolhaas et al., 1990; Dabbs et al., 1995) and social status (Mazur and Booth, 1998; Josephs et al., 2003). On the other hand, sexual behavior, competition for status or fighting can alter endocrine levels (Mazur and Lamb, 1980; Elias, 1981; Carmichael et al., 1994).

Previous research in primates and humans suggests that high levels of testosterone promote behaviors intended to enhance one's status over other individuals and to climb up the social hierarchy. According to the biosocial model of status (Mazur, 1985), status defense can overtake a form of dominance or aggression. An individual is *dominant* if its intent is to gain or defend high status over another member of its species. An *aggressive* individual will have the intent to inflict physical and psychological injury on a conspecific. Sometimes dominant behavior takes the form of aggressive or antisocial behavior such as violence or law breaking. However, the distinction between dominance and aggression is particularly important in humans, where dominance is often asserted without any intent to cause injury. For instance, Ehrenkranz et al. (1974) showed that both, aggressive prisoners and dominant, but non-aggressive prisoners had a significantly higher level of plasma testosterone as compared with non-aggressive and low dominance prisoners.

### Measuring antisocial behavior in the lab

In the laboratory, socio-economic games are widely used to study non-aggressive anti-social behavior. Socio-economic games are social decision-making trials simulating real-world strategic interactions (Camerer, 2003). Involved individuals make monetary choices based on an interdependent pay-off matrix. The two bargaining partners are given a set of rules and they face limited information since they are confronted with uncertainty about the other's intentions (see below for details). Importantly, the individuals' choices alter not only their own outcome, but also the outcome of the other, allowing the researcher to study game-theoretical constructs such as fairness, reputation building and status defense. While prosocial behavior or altruism are often the target dependent variables of investigation, recent attempts have been made to use socio-economic games for measuring anti-social or assertive behavior as in the tendencies to punish and retaliate (Falk et al., 2005; Nikiforakis, 2008; Yamagishi et al., 2012). The public goods game is a stylized model of situations that require cooperation to obtain socially beneficial outcomes in the presence of incentives for free riders. By using this game, Herrmann et al. (2008) showed that antisocial punishment exists in different participant pools around the world. The punishment of unfair behavior such as free riding may arise from negative emotions that are evoked through feeling exploited. Accordingly, emotions such as anger or moral disgust make individuals disregard the immediate consequences of their behavior, allowing them to preserve a reputation over time as someone who is reliably committed to this behavior (Yamagishi et al., 2009).

The ultimatum game (UG) allows to study the tendency to punish unfair behavior (negative reciprocity) in the responder. The UG (Güth et al., 1982) is a two stage socio-economic game in which a proposer is given a monetary endowment, which he can split and share with a responder. Only if the responder accepts, both players receive their share according to the proposer's split. Thus, the proposer has the power to postulate an ultimatum to the responder. Economists argue that negative reciprocity reflects altruistic punishment (Fehr and Gächter, 2002), since the rejecting individual is sacrificing own resources. However, recent studies suggest that self-interest, in terms of status defense, plays a substantial role in decisions to reject unfair offers (Yamagishi et al., 2012; Kaltwasser et al., 2016). According to the above mentioned biosocial model of status (Mazur, 1985), individuals with high levels of testosterone should be more likely to retaliate, e.g., have a greater desire to harm those who committed unfair acts. While most studies focused on the responder behavior in UG in order to quantify negative reciprocity as the tendency to reject unfair offer, for each participant, we obtained data in both roles of the UG—as proposer and responder. This “dual” version of the UG is valuable not only in order to obtain preferences for fear of punishment (strategic behavior) in the proposer data, but also in order to study whether the assigned role affects cooperation behavior in general. For example Brañas-Garza et al. (2006) investigated behavior in a dual UG with illiterate gypsies in Vallecas, Madrid, acting as both proposer and responder. In this set-up, the responder's acceptance of a zero offer was not a rare case, but the modal value, and 97% of the subjects proposed an equal split in the role of the proposer.

### Ratio of second-finger-length to fourth-finger-length (2D:4D)

The scientific study of the impact of sex steroids on brain and behavior has been separated into activational and organizational effects. Activational effects are temporal and occur throughout life depending on current hormone levels. Organizational effects are permanent and mainly occur in two phases: early in development when most neural structures are formed and during adolescence (Phoenix et al., 1959). However, empirical evidence speaks against a rigid dichotomy between both classes of effects (Arnold and Breedlove, 1985). Studies provided by the animal model suggest that organizational hormones may prime the brain by changing its responsivity to hormones that are present later in life (Clark and Galef, 1998).

There is some evidence for prenatal organizational effects of sex steroids (for a review see Auyeung et al., 2013). For example, twin studies have been conducted following the assumption that females from pairs of opposite-sex twins are exposed to higher levels of prenatal testosterone compared to same-sex twins. While free circulating testosterone levels were not yet systematically related to different personality traits, a sex difference in aggression proneness has been observed. Opposite-sex girls of the twin dyad studied show a more masculine pattern of aggression proneness than same-sex girls (Cohen-Bendahan et al., 2005a).

Furthermore, females with Congenital Adrenal Hyperplasia (CAH), a genetic disorder which causes excessive androgen levels during early development, show a masculinization of their behaviors, for example in playing (Hines, 2003) and spatial navigation (Hampson et al., 1998), as well as with respect to cognitive abilities (Resnick et al., 1986) and personality traits (Berenbaum and Resnick, 1997; Mathews et al., 2009). The studies with CAH participants suggest that differences between males and females are due to androgens as testosterone, but they are less informative about the role of androgens in producing typical variations (Cohen-Bendahan et al., 2005b).

Similar to persons with CAH, individuals with androgen insensitivity syndrome, who have androgen levels typical for males and XY generic structure but do not react to androgens due to dysfunction of androgen receptors, show a higher ratio of second-finger-length to fourth-finger-length (Berenbaum et al., 2009; van Hemmen et al., 2017; 2D:4D). Therefore, 2D:4D with smaller values is considered to mark stronger prenatal testosterone exposure (Manning et al., 1998) and it is taken to be a static indicator of prenatal testosterone in normally developing humans.

This interpretation is partly endorsed by similar timing of both, the prenatal digit development and the highest prenatal testosterone levels (Vaillancourt et al., 2012), and the relation of sex hormones and bone growth established in research on mammals (Kondo et al., 1997). One of the most cited papers providing evidence for the usability of 2D:4D as an indicator of organizational effects of sex steroids reported a negative correlation of right-hand 2D:4D with the ratio of testosterone and estrogen in the amniotic fluid mid gestation (Lutchmaya et al., 2004). However, this finding should be interpreted with caution. The reason is first the used methodology (Hollier et al., 2015; Yeung and Tse, 2017) and second, the fact that the relation of sex-hormone levels in amniotic fluid with levels of sex-hormone in the fetus blood are not well-established (Cohen-Bendahan et al., 2005b). When sex steroid levels were measured in umbilical cord, no systematic relation to 2D:4D could be established (Hollier et al., 2015; Mitsui et al., 2016), which might also result from differing levels of steroid hormones during prenatal development.

2D:4D shows a moderate but stable sex difference (Hönekopp and Watson, 2010) that develops early during fetal development and individual scores remain stable across development. Sex differences in 2D:4D are noticeable already at the end of the first trimester of prenatal development (Malas et al., 2006), but become relatively stable after 5 years of age and do not change during puberty. There are three stages during development in boys when testosterone reaches levels similar to those in adult men: (a) during 10th to 18th week of prenatal development, (b) 1–2 weeks after birth, and (c) from 8 weeks until 4–6months of age (McIntyre, 2006). Thus, based on these findings, 2D:4D might be considered an indicator of perinatal organizational effects. Interestingly, circulating steroid levels are unrelated to 2D:4D, suggesting that relationships between 2D:4D and target variables reflect effects of prenatal testosterone exposition (Hönekopp et al., 2007). Notwithstanding, evidence regarding the association between 2D:4D and trait variables, such as personality or facets of socio-economic decision-making is mixed.

A meta-analysis comprising 64 samples with *N* = 6,617 females and males (Hönekopp and Watson, 2011) found no evidence for 2D:4D predicting aggression at different levels of behavior, ranging from physical and verbal aggression to anonymous contacts. The study only revealed a small negative association (*r* = −0.06) between 2D:4D and aggression in males, which was absent in females. No evidence was found that either hand would predict aggression better than the other—a finding that is corroborated with other target variables such as athletic prowess (Hönekopp and Schuster, 2010). Apicella et al. (2008) showed in a sample of *N* = 98 men that risk-taking in an investment game correlates positively with salivary testosterone levels (*r* = 0.29) and facial masculinity (*r* = 0.27), with the latter being a proxy for pubertal hormone exposure (see section on WHR below). 2D:4D on the other hand did not correlate with risk preferences.

Another personality trait that has been studied in conjunction with testosterone is assertiveness, the quality of being self-assured and confident. Depending on the scale used to measure assertiveness, this trait is correlated with aggression or status-imposing behavior (Buss and Perry, 1992; Yamagishi et al., 2012). While Hampson et al. (2007) found lower 2D:4D ratios to be associated with increased aggressiveness and sensation seeking, no such relationship was present for assertiveness. The absence of a relationship between 2D:4D (for both sexes and hands) and assertiveness was further confirmed by a study with a larger sample of 491 men and 627 women (Voracek, 2009).

Studies relating 2D:4D to socio-economic bargaining suggest that the broader picture of the relationship between static markers of the “status-hormone” with prosocial vs. antisocial or status-enhancing behavior is complex. Recent evidence suggests a non-monotonic, i.e., u-shaped, impact of prenatal testosterone exposure on altruism in the sense that individuals with both, high and low digit ratios give less than individuals with intermediate digit ratios (Brañas-Garza et al., 2013; Galizzi and Nieboer, 2015). Moreover, a study administering testosterone to women showed a substantial increase in *fair* bargaining behavior in the UG (Eisenegger et al., 2010). Interestingly, participants who believed that they received testosterone (regardless of whether they actually received it) showed more unfair behavior than those who were treated with placebo—providing evidence for the power of folk wisdom on participant's expectations about testosterone as a status or even aggression inducing hormone. A later publication commenting the latter study suggests that static marker of prenatal testosterone may interact with administered testosterone, in that social cooperation increases after testosterone administration but only in participants with low levels of prenatal testosterone measured by right hand's 2D:4D (van Honk et al., 2012).

### Facial width-to-height ratio (WHR)

Another characteristic that has been related to testosterone is the WHR, that is, the face width divided by upper-face height. Research on this topic stemmed mostly from the observation that WHR is a sexually dimorphic face characteristic (Weston et al., 2007; Carré and McCormick, 2008), although a meta-analyses lead to equivocal conclusions regarding the existence of this dimorphism (Geniole et al., 2015; Kramer, 2017). Taking the finding into account that WHR dimorphism develops during adolescence (Weston et al., 2007), and because boys' craniofacial growth has shown to be enhanced by testosterone administration (Verdonck et al., 1999), WHR was suggested as a proxy for organizational hormonal effects in adolescence (Carré and McCormick, 2008). However, research on how changes in testosterone levels during adolescence are related with WHR gave equivocal results (Hodges-Simeon et al., 2016; Welker et al., 2016). Similar to 2D:4D, WHR showed no relationship to circulating testosterone levels in adulthood (Bird et al., 2016). As expected based on the idea that WHR is an organizational hormonal effects' proxy specifically of adolescence, adult WHR showed no relation to umbilical testosterone levels (Whitehouse et al., 2015). In the same study, WHR also showed no relationship with 2D:4D (ranging between *r* (*N* = 75) = −0.22, n.s., for female left hand, to *r* (*N* = 82) = 0.11, n.s., for male right hand). To our knowledge, this is the only research inspecting the relationship of 2D:4D and WHR.

Two meta-analyses were recently published on the relation of WHR to aggression (Haselhuhn et al., 2015) and threatening and dominant behaviors (Geniole et al., 2015). The first study included only men and a narrower range of behavior and published papers. These studies concluded a weak, albeit significant relation of WHR and status-enhancing behavior in men, with the effect size ranging between *r* = 0.11 and 0.16., *p* ≤ 0.01. For women, the effect was significant only in case of dominant behavior. Different related psychological constructs have been proposed as mediators between WHR and aggressive behavior, such as fearless dominance (Geniole et al., 2014; Anderl et al., 2016) and psychological sense of power (Haselhuhn and Wong, 2011).

The socio-economic choices mostly fit into this pattern, with men having higher WHR exploiting others' trust more in a trust game (Stirrat and Perrett, 2010) and cheating more in a lottery (Haselhuhn and Wong, 2011; Geniole et al., 2014). However, Stirrat and Perrett (2012) demonstrated that WHR is not necessarily related with antisocial behavior. In their experiment, WHR predicted higher cooperation, leading to the player's individual loss, when it benefited their group at the expense of an out-group. This might be a strategy to enhance one's status in the in-group, and is in accordance with the postulated relation of testosterone and status. Moreover, it reflects behavior in line with the male warrior hypothesis which suggests that men have a stronger tendency to treat in-group members benevolently and out-group members malevolently compared to women (Van Vugt et al., 2007).

### Current study

In the light of the above reviewed studies, evidence on the relationship of testosterone with facets of socio-economic decision-making such as status defense are provided by two sources: First, there is research on acute effects of testosterone and human social decisions. That research includes studies administering testosterone and investigating its consequences on social decisions by using laboratory paradigms (for a review see Bos et al., 2012). Since testosterone not only affects behavior but it also responds to it, it can also serve as the dependent variable in experimental procedures where social interaction parameters, such as status, are modulated and testosterone is measured and an outcome (Carney et al., 2010). Second, stable trait-like dispositions with regard to testosterone can be the matter of study—including static markers of testosterone, which are consequences of developmental differences in testosterone exposition. In the current study we investigate the association of such static markers of testosterone with facets of socio-economic decision making in a typically developing population of young adults. As far as we know, this is the first study to relate WHR and 2D:4D to facets of socio-economic decision making within one statistical model, therefore allowing to estimate the shared variance of different markers of exposure to testosterone during early stages of development.

#### Hypotheses

Based on the reviewed literature on testosterone and facets of socio-economic decision making, we expected participants with lower 2D:4D to show increased assertive and less prosocial behavior. If unfair UG offers are seen as provocations, then individuals with stronger prenatal testosterone exposition may be more prone to reject such offers.

Regarding WHR we hypothesized that individuals with wider faces show more masculinized behavior—reflected in more assertive and less prosocial behavior.

Since the evidence for gender effects in the associations between both static markers of testosterone and the target variables is rather inconsistent, we modeled the relationships separately and together for both genders.

More recent literature discussed above suggest an inverted U-shaped relationship between prenatal testosterone exposition and prosocial behavior. We thus tested whether individuals with lower vs. higher 2D:4D show less prosocial behavior as compared with persons with intermediate 2D:4D. Following the same argumentation, a U-shaped relationship may be predicted for rejections in the Ultimatum Game indicating negative reciprocity due to provocative behavior.

## Methods

### Participants

The reported data stems from a sample of 84 females and 91 males (*N* = 175) who took part in a larger study investigating socio-emotional processes and abilities. Participants gave consent of their pictures being used for further investigations (Kaltwasser et al., 2016). The mean age of this sample was 27.62 (*SD* = 5.4). Participants were recruited through the university's participant pool and public announcement in newspapers as well as on local websites. The study conformed to the guidelines of the ethics committee of the Department of Psychology, Humboldt-Universität zu Berlin. All experiments were in accordance with the Declaration of Helsinki. The protocol was approved under the approval number 2013-17. All participants provided written consent before starting the experimental procedures. They received a compensation of 8 € per hour and were informed that they could win further money during the UG, depending on their choices. Each participant received an additional amount of 5 € as payout from UG. Seventy-seven percent of the participants had completed German high school of which 35% had a university degree. Forty-six percent of the sample where still studying while the rest was working full-time or unemployed (16%).

### Procedure

The experiment consisted of two sessions. During the behavioral session that lasted 2 h, participants completed computerized self-report measures of personality and fairness preferences, as well as several ability measures of face and object cognition, which are not analyzed for the scope of this paper. All questionnaires were programmed in Inquisit software (Inquisit 4.0.0.1, 2012; Millisecond Software, Seattle, WA), and responses were given via computer mouse. In the laboratory session, taking place 1–2 weeks after the behavioral session, participants were photographed and 2D:4D measurements were acquired by means of a photocopy machine. Additionally, they played the UG as proposer and responder. During data acquisition of the UG, in the responder condition we also measured the participants' EEG. Electrophysiological measures are however not the scope of this paper.

#### Assertiveness

We applied the assertiveness scale of the German Inventory of Personality Styles and Disorders (Persönlichkeits-Stil-und-Störungs-Inventar) (Kuhl and Kazén, 2009). The scale consists of 10 items (α = 0.82) measuring the tendency to impose oneself onto others and the tendency to defend ones' status. This tendency may extend to ruthless and antisocial behavior. A sample item is “*If others want something which I need, I normally prevail*.” Responses are given on four-point Likert scales (disagree strongly, disagree somewhat, agree somewhat and agree strongly). For the analyses, we formed three parcels of three to four items each based on the underlying motivation for assertiveness as reflected in the content of the item (aggressive, egoistic, or assertive behavior).

#### Social value orientation (SVO)

The magnitude of concern people have for others can be measured by a six-item questionnaire (α = 0.89), where participants indicate how they would share resources with an anonymous stranger (Murphy et al., 2011). Each item is a resource allocation over a continuum of joint payoffs. For example, the participant has to choose a value x_self_ between 50 and 100, knowing that the anonymous partner will get x_other_ = 150–x_self_. According to the pay-off structure, the participant is assigned a continuous value of social orientation, which can be categorized to competitive, individualistic, prosocial and altruistic. Previous research indicates that SVO is a valid predictor of the cooperative tendency in social dilemmas (Bogaert et al., 2008; Balliet et al., 2009). In the analyses, we formed three parcels out of two SVO items each to serve as indicators for the latent factor of prosociality next to the indicator of total offers in the responder part of the Ultimatum Game (see next section).

#### Ultimatum game (UG)

**Figure 1:**
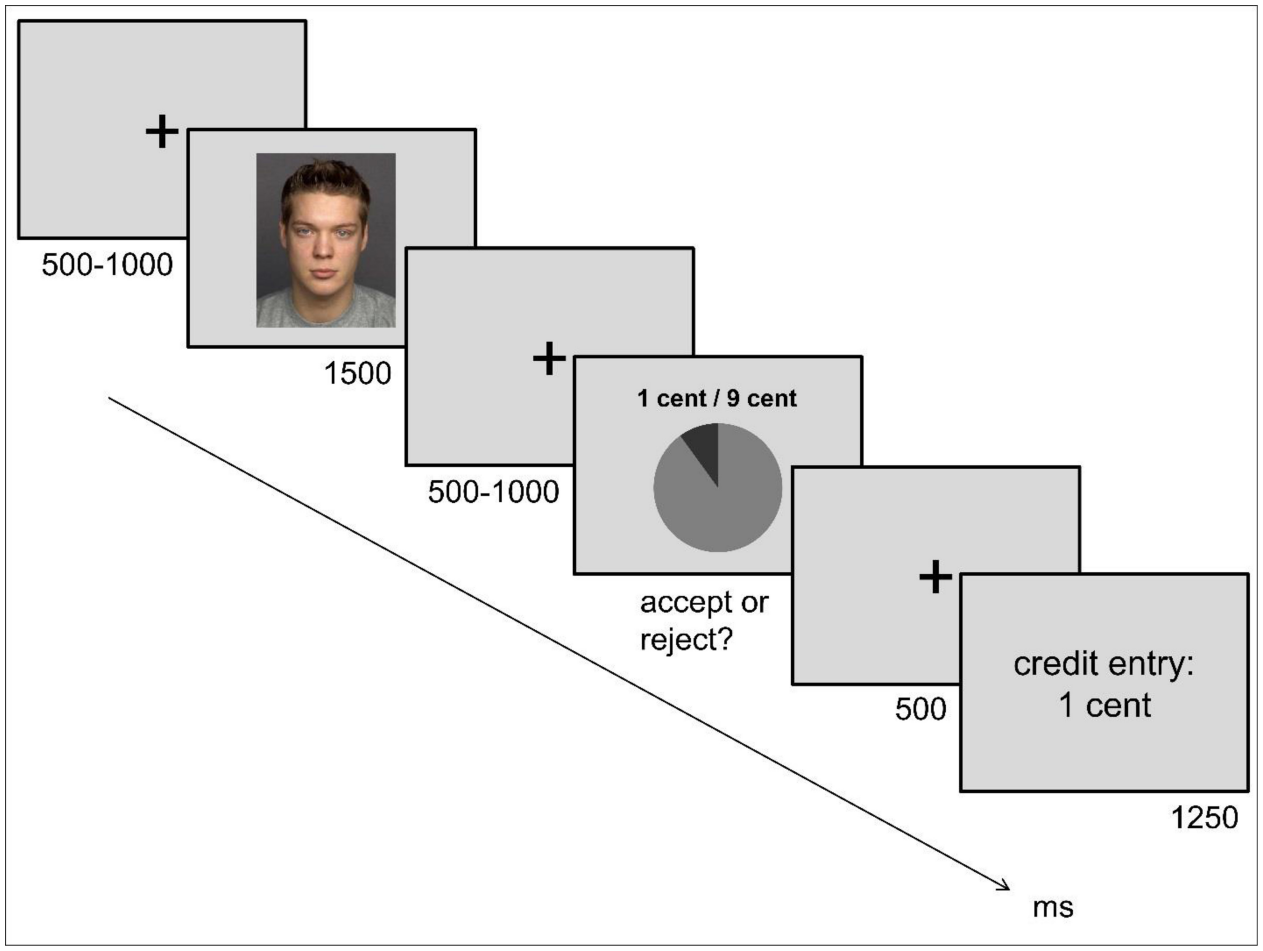
Trial Scheme of the Ultimatum Game. Each trial started with a fixation cross shown for a variable time of 500–1000 ms, followed by a photograph of a proposer for 1500 ms, and another fixation cross presented for 500–1,000 ms; then, participants received an offer about splitting 10 cent which they had to accept or reject via button press. Afterwards, a fixation cross was presented again for 500 ms. Participants received feedback about the sum booked to their account before the next trial started after 1,250 ms. Portrait taken from Ebner et al. (2010) for which the depicted individual gave consent to be displayed in research-related publications.

Upon arrival to the laboratory, participants were introduced to the rules of the UG, informing them that they would play with other participants, which would require having their picture taken. Moreover, participants were asked to play the proposer in the UG, making 12 offers on a query sheet. In each offer, the participant could divide 10 cents into two shares: one for her/him and one for the other player. There were three predefined proposals: 9/1 (nine for the proposer, one for the responder), 7/3 and 5/5. Participants were informed that these offers would later be presented to other players together with their picture. They were instructed that the other player could then decide whether to accept or reject each offer. Participants were told that they would receive the corresponding amount of money if the offer was accepted by the responder. After providing their offers on a sheet, participants played the computerized version of the UG in the role of the responder while EEG was recorded (288 trials). They were explained that they would receive monetary offers made by six previous participants, but the actual offers came from six pseudo-proposers (50% females). Due to the EEG methodology whose data is published elsewhere (Kaltwasser et al., 2016) we required an experimental protocol of the UG which allows for a specific offer distribution and high signal-to-noise ratio, e.g., many trial repetitions. Hence, it was necessary to deceive the participants in the origin of the proposals they saw. These proposers were represented by portraits taken from a standardized stimulus set, the FACES database (Ebner et al., 2010). We included portraits of the proposer prior to the offers in order to create a social bargaining situation, since previous work suggests that social cues affect cooperation behavior (Haley and Fessler, 2005). The responder version of the UG comprised trials with fair (5/5), slightly unfair (7/3), or highly unfair (9/1) offers which were paired with the same proposer identities, so that the participant could learn over the course of the experiment, that two proposers always made fair offers, two always made unfair offers, and two made mixed offers. The rejection rates of unfair offers for each of the three experimental blocks served as indicators for the latent factor of negative reciprocity. A typical trial of the responder version of the UG with an unfair offer is depicted in Figure 1.

#### Facial photographs

Full frontal facial photographs were taken of all participants without glasses or head wear with a Panasonic HDC-SD707 on a tripod in front of a gray background. The distance between the camera and the subject was kept consistent with 1.5 m. The portraits were preprocessed and cut into rectangular facial images of the same size (e.g., removing the presence of the neck and the remaining space above head) using Photoshop. Pictures of the participants who gave consent of their pictures being used in further studies were used for the analyses reported below. Eighty-six percent of the sample of Kaltwasser et al. (2016) agreed and their data is reported here.

#### WHR measurement

Two raters independently measured facial width and height on the full frontal photographs using ImageJ 1.48 software (Schneider et al., 2012). Width was defined as the distance between the points on the picture where ears and face meet. Height was the distance from the point where the brow touches the root of the nose to the highest point of the lips (Weston et al., 2007; Carré and McCormick, 2008).

#### 2D:4D measurement

The ratio of second-finger-length to fourth-finger-length was acquired for the left and right hand independently. A see-through foil with a printed standard ruler was placed on the scanner for each participant (in accordance with Kemper and Schwerdtfeger, 2009). Before scanning, the proximal crease was marked with a water-soluble marker as to ease the determination of ventral proximal crease (in accordance with Voracek et al., 2007). Participants were instructed to press lightly with both hands at the same time. The experimenter verified that participants followed the instruction and checked that their hand position was in accordance to the guidelines provided by Mayhew et al. (2007). As suggested by Hiraishi et al. (2012), white cloth was put on the hands by the experimenter in order to achieve more contrast and an easy determination of points on the scanned pictures. Scans were made using HP Scanjet 7650 and the resolution was kept standard. Two raters with previous experience with 2D:4D measurement independently measured digit lengths using specialized open source software AutoMetric (DeBruine, 2004).

### Data analysis

Latent factors of 2D:4D, WHR, prosociality, negative reciprocity and assertiveness, along with their mutual relationships were estimated in measurement and structural models using structural equation modeling conducted with the lavaan package (Rosseel, 2012) in the R software for statistical computing (R Core Team, 2017). For testing specific relationships due to sex between those latent variables, multi-group structural equation models (e.g., Little et al., 2007) were fitted using the same software. Structural equation models (SEM) can be used to test theories on linear relationships between multiple psychological entities by explicitly accounting for measurement error and the specificity of the measurement method (Bollen, 1989). SEMs estimate latent variables based on their measured, observable indicators. The basic idea behind latent variables is that all psychological measurements are error prone and contain measurement method specificity. For example, the measured values of 2D:4D from hand image scans by two different raters will not completely overlap. Using the multiple rating values provided by different raters as indicators of a latent variable to be estimated on the basis the indicators' covariances allows taking rater specific measurement error into account. Thus, latent variables are quantifying the true score variance of 2D:4D, WHR and the traits to be studied in the present work. The quality of SEMs can be assessed by multiple formal statistical tests and fit indices: Chi-square statistics, the root mean square error of approximation (RMSEA, should be lower than 0.08), standardized root mean square residual (should be SRMR < 0.08) and the Comparative Fit Index (should be CFI>0.95; see e.g., Bollen, 1989 for details).

For testing the non-linear relationship between 2D:4D with prosociality and with negative reciprocity, we used an exploratory method called Local Structural Equation Modeling (LSEM; Hildebrandt et al., 2016). This method allows estimating an SEM along the values of a moderator. Because we are interested to explore curvilinear relationships between 2D:4D and prosociality, we aim to estimate the measurement models of prosociality and negative reciprocity along continuously sampled values of 2D:4D within its possible range of measured values. The LSEM modeling approach allows to investigate whether the mean of the latent prosociality and negative reciprocity factors are different across varying values of 2D:4D. Based on LSEM estimates, the latent factor means of prosociality and negative reciprocity can be plotted along the values of 2D:4D. Thus, for the present research the range of the 2D:4D left vs. right hand variables was taken as a continuous scale along which the latent factor mean of prosociality and negative reciprocity may vary, following an inverted U-shaped or U-shaped curve, respectively (see hypotheses on non-linear relations above). We thus provide parameter plots estimated by LSEM to illustrate how average prosociality and negative reciprocity varies along the measured values of 2D:4D. In summary, these gradients visualize curvilinear relations between prosociality and negative reciprocity, respectively, with the 2D:4D measurements (see Hildebrandt et al., 2016 for details on LSEM). LSEM was conducted with the sirt package in R (Robitzsch, 2015).

## Results

To test our hypotheses, we run a series of measurement and structural models including latent variables representing organizational effects of hormones measured by estimations of (1) 2D:4D (left and right hand) provided by two different raters and (2) of WHR estimated by two raters as well. Furthermore, (3) prosociality, (4) negative reciprocity, and (5) assertiveness was modeled based on multiple measured behavioral indicators. Thus, in a first step we estimated a measurement model of 2D:4D and WHR, including three latent factors because 2D:4D has been measured on the right as well as on the left hand side by two different raters. Consequently, there are two indicators (provided by two different raters) for each of the three latent variables representing prenatal and pubertal organizational effects of hormones. In a second step we aimed to establish a measurement model for the behavioral indicators of prosociality, negative reciprocity and assertiveness. We estimated the latent factor of prosociality by means of three parcels of SVO responses (see above) and a further indicator of total offers in the proposer part of the Ultimatum Game. Negative reciprocity as a latent variable is measured by rejection rates of unfair offers in three independent experimental blocks and assertiveness is reflected by three indicators of different underlying motivations for assertiveness (aggressive, egoistic, or assertive behavior; see also task descriptions in the method section above). Third, the two measurement models were related to each other in a structural equation model of hormone-behavior relations. Fourth, the structural model was simultaneously estimated for males and females using the well-established technique of multiple group modeling. As customary in multiple group analyses (see Little et al., 2007), the sex specificity of hormone-behavior relations was tested after establishing measurement invariance across sex. This is to ensure that the factors can be interpreted as isomorphic (equivalent) for males as compared with females. If indicators are measuring the latent variables with the same precision—thus, factor loadings would be equal for males and females—we could conclude that the association between hormones and behavior are statistically and substantially comparable across sex because the meaning of the factors are equivalent. Last, we tested a curvilinear association between hormones and prosocial behavior vs. negative reciprocity in the whole sample to investigate whether their relationship is rather inverted U-shaped vs. U-shaped in case of negative reciprocity, and not linear (see discussion above and the data analyses section for details on the LSEM procedure).

### Measurement model of 2D:4D and WHR

**Figure 2:**
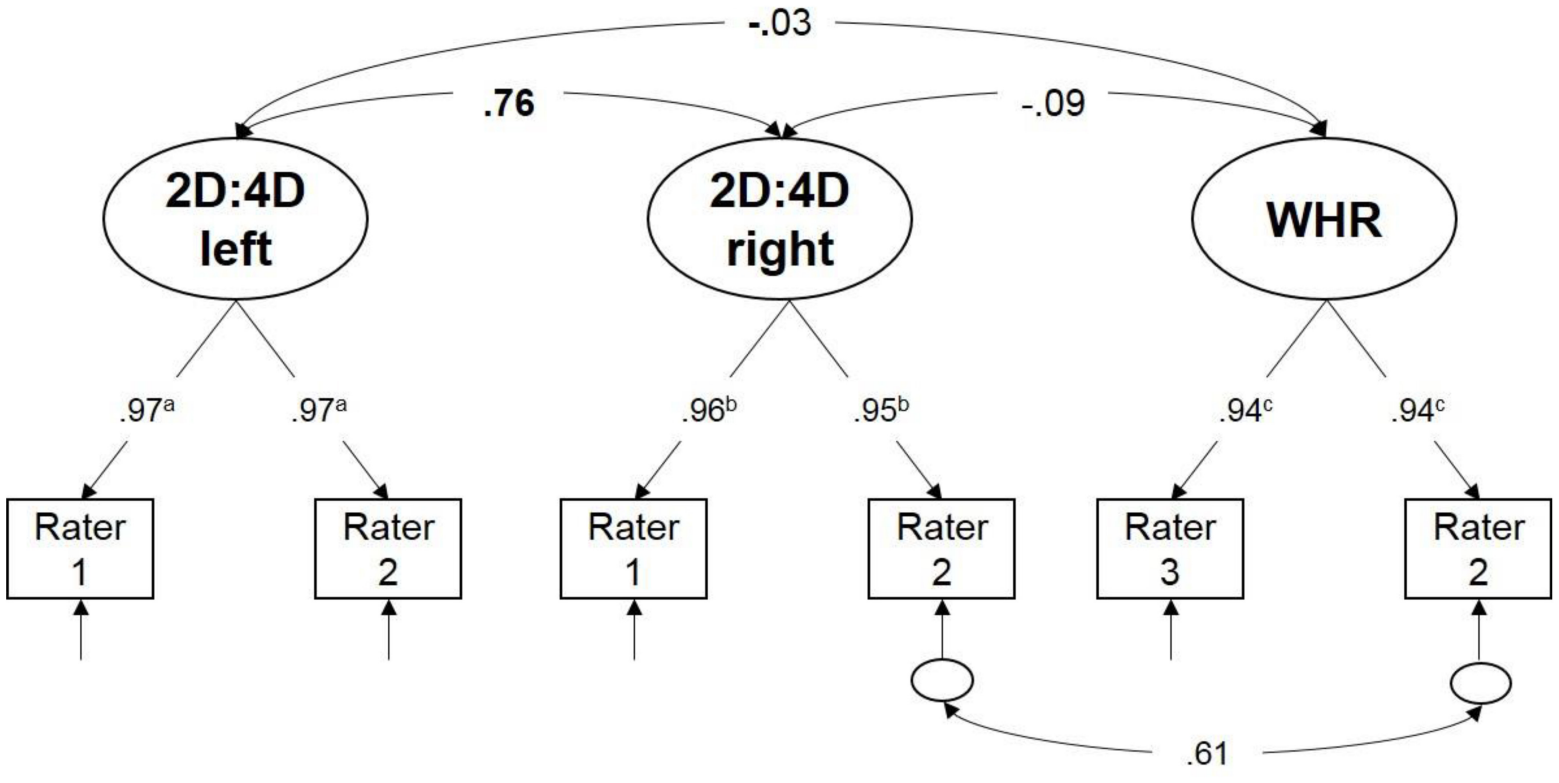
Schematic representation of the measurement model of prenatal and pubertal organizational effects of hormones. Rectangles represent measured variables and circles are used to depict latent variables. 2D:4D at the left and the right hand was measured by two different raters. Rater specific values are used as measured variables to estimate latent variables that represent prenatal and pubertal organizational effects accounted for measurement error due to the rater. Unidirectional path represent factor loadings and bidirectional path are used for depicting correlations. Short arrows (with a small circle) represent residual variance (non-reliability of a measured variables). For simplicity, we use only arrows to indicate error variance if there is no residual correlations between residuals. 2D:4D left—left hand 2D:4D estimation taking rater induced measurement error into account; 2D:4D right—right hand 2D:4D estimation; WHR—facial width-to-height ratio estimate taking rater induced measurement error into account; a, b, c indices on factor loadings are used to indicate that non-standardized loadings were fixed to equality within factors; standardized loadings are depicted in the figure. Significant relationships between latent factors at *p* < 0.05 are written bold.

2D:4D at the left and right hand and WHR were estimated by three different raters (see Figure 2). These ratings for each person included in the final sample were used as indicators for measuring three latent factors–2D:4D left, 2D:4D right and WHR—to be established in the measurement model of organizational effects of hormones. The model depicted in Figure 2 fitted the data very well: χ^2^(8) = 3.79, *p* = 0.88, CFI = 1, RMSEA = 0.00, SRMR = 0.02. Because only two indicators were available for each factor, their non-standardized loadings were fixed to equality within each factor (note that standardized loadings are depicted in Figure 2). The model fit was excellent in spite of equality constraints on the factor loadings. High standardized factor loadings depicted in Figure 2 suggested that 2D:4D and WHR measurements were highly consistent across raters based on the above described measurement procedure. Latent factor correlations revealed that 2D:4D is not related with WHR, whereas left and right hand 2D:4D are substantially (*r* = 0.76), but not perfectly correlated. Having the same rater across different indicators led to a correlated error between Rater 2 of right 2D:4D and WHR (see Figure 2) which needs to be included in order to achieve good model fit.

### Measurement model of prosociality, negative reciprocity and assertiveness

**Figure 3:**
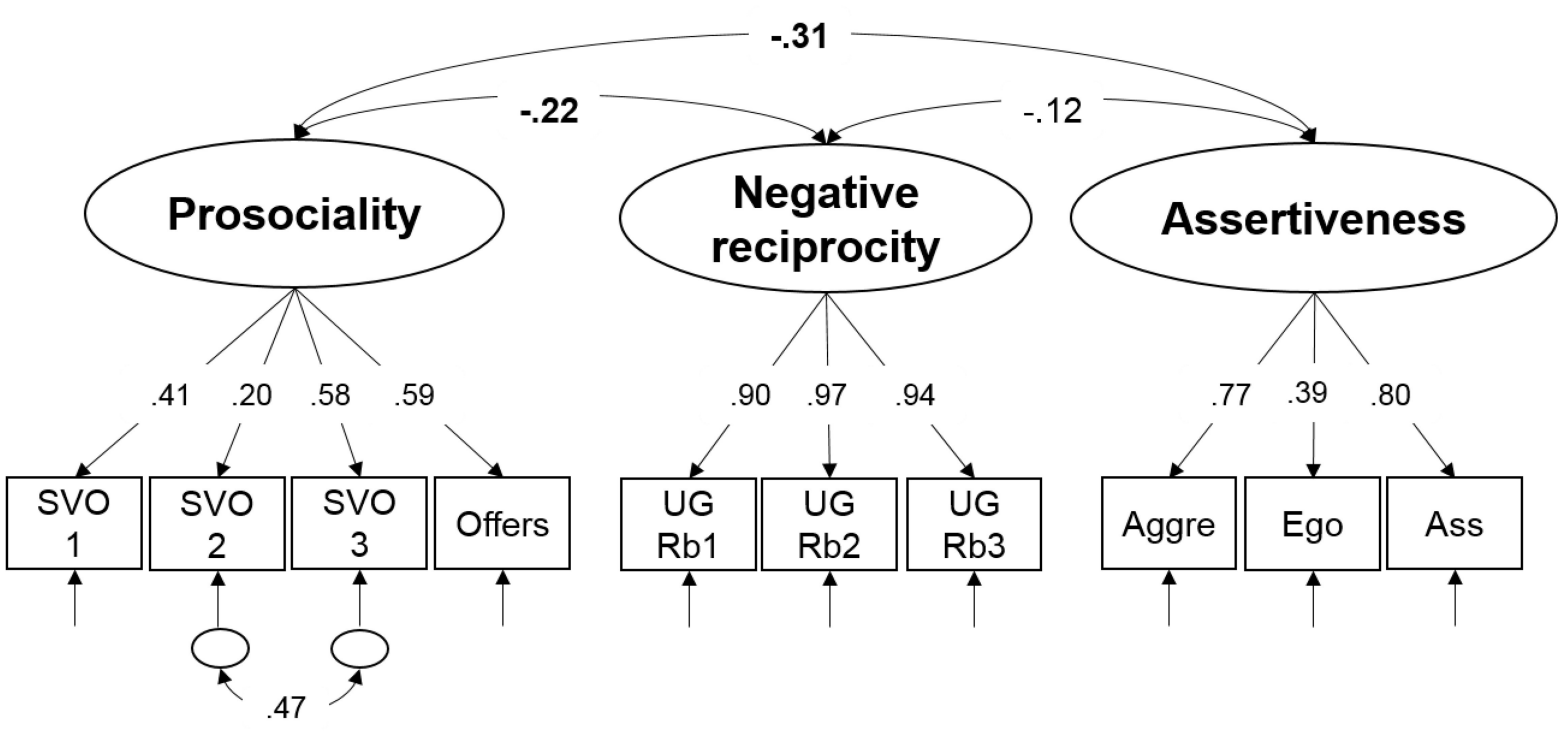
Schematic representation of the measurement model of prosociality, negative reciprocity and assertiveness. See the caption of Figure 2 explaining the general conventions of the graphical language visualizing latent variable models. SVO, Social value orientation; UG, Ultimatum Game; Rb1, Rejection of unfair offers in bloc 1; Rb2, Rejection of unfair offers in bloc 2; Rb3, Rejection of unfair offers in bloc 3; Aggre, Aggressive; Ego, Egoistic; Ass, Assertive. Significant relationships between latent factors at *p* < 0.05 are depicted in bold.

In the second measurement model displayed in Figure 3, behavioral indicators described in the method section were used to estimate three latent factors—prosociality, negative reciprocity and assertiveness. The measurement model, including one theoretically expected residual covariance between indicators of SVO due to similar pay-off structures, had a very good fit to the data: χ^2^(31) = 34.63, *p* = 0.30, CFI = 0.99, RMSEA = 0.03, SRMR = 0.04. Standardized factor loadings (see Figure 3) were all significantly different from zero and were substantial in their magnitude. Prosociality showed a small negative association with negative reciprocity and assertiveness, whereas the relation between assertiveness and negative reciprocity did not reach statistical significance.

### Structural model of organizational hormonal effects and behavior

**Figure 4:**
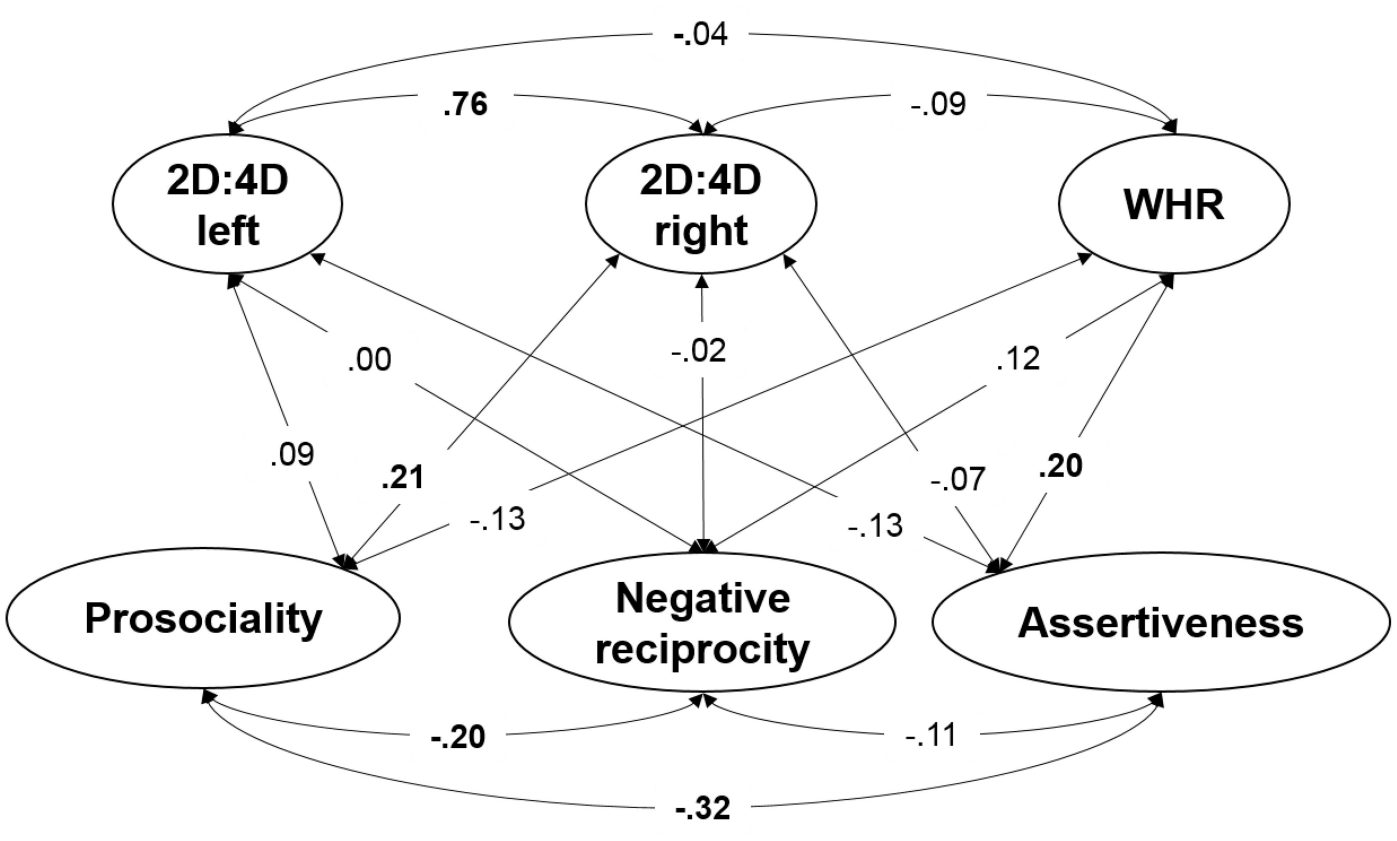
Schematic representation of the structural model testing the relationship between prenatal and pubertal organizational effects of hormones and prosociality, negative reciprocity and assertiveness. Significant relationships between latent factors at *p* < 0.05 are depicted in bold. See the caption of Figure 2 explaining the general conventions of the graphical language visualizing latent variable models. Note that for simplicity Figure 4 only depicts the latent variables. The measurement models of each latent variable included in this structural model was the same as shown in Figure 2 for the latent variables representing prenatal and pubertal organizational effects and Figure 3 for the latent variables quantifying prosociality, negative reciprocity and assertiveness.

To estimate the relationship between prenatal and pubertal organizational effects of hormones and prosociality, negative reciprocity and assertiveness, the two measurement models established above were related to each other in a full structural equation model. The measurement models were completely equivalent to those described above. All bivariate relationships between latent factors were estimated. The structural model also had an excellent fit to the data: χ^2^(90) = 90.23, *p* = 0.47, CFI = 1, RMSEA = 0.00, SRMR = 0.04. The correlations between 2D:4D, WHR and trait factors are provided in Figure 4. There was no association between organizational effects of prenatal and pubertal hormones and traits, except for a small positive association between WHR and assertiveness, suggesting that persons with higher facial width-to-height ratio are more assertive. A further positive association prevailed between right hand 2D:4D and prosociality, suggesting that persons with higher 2D:4D are somewhat more prone to prosocial decisions.

### Sex differences in organizational effects of prenatal and pubertal hormones and behavior

As discussed above, in the light of the literature, sex differences are expected regarding hormone-behavior relationships depicted in Figure 4. As a prerequisite of comparing association in a structural equation models across groups, measurement invariance needs to be tested, because the test assures the meaning of the latent variables to be equivalent across groups. Model parameters at the level of latent variables are only comparable across groups if measurement invariance can be confirmed (see Little et al., 2007).

Measurement invariance implies a stepwise test of increasingly restricted models. In a first step a model with freely estimated parameters will be inferentially compared with a model in which factor loadings are fixed to equality across sex groups. The second step includes further cross-group equivalence restriction on intercepts. The results of these invariance tests are displayed in Table 1. Whereas, factor loadings are invariant for females and males, the intercepts seem to be biased for sex. Such an outcome is indeed comprehensible bearing in mind the existing sex differences in the variables quantifying hormonal influences and the high inter-rater consistency. We were however not interested to compare factor means in the multigroup model, but to investigate whether the hormone-behavior relationship differed for females and males. For group-comparison regarding relationships between latent variables invariance of factor loadings in a necessary and sufficient condition. Since factor loading invariance across sex was demonstrated for the present data (see Table 1), comparisons of hormone-behavior relations are possible and sound. However, multiple group modeling of the structural model depicted in Figure 4 revealed no statistically substantial hormone-behavior associations neither in the group of females, nor males. The magnitudes of the relations were comparable across females and males and somewhat lower as compared with those displayed in Figure 4.

**Table 1:** Results of invariance testing across sex.

**Model**	**χ^2^**	***df***	**CFI**	**RMSEA**	***Δχ*^2^**	**Δ*df***
Configural invariance	203.54	177	0.986	0.042	–	–
Weak (metric) invariance	230.27	193	0.981	0.048	26.72	16
Strong (scale) invariance	284.59	209	0.958	0.069	54.31^*^	16

* *p < 0.01; CFI, Comparative Fit Index; RMSEA, Root Mean Square Error of Approximation*.

### Curvilinear relations between organizational effects of prenatal and pubertal hormones, prosocial behavior, and related traits

**Figure 5:**
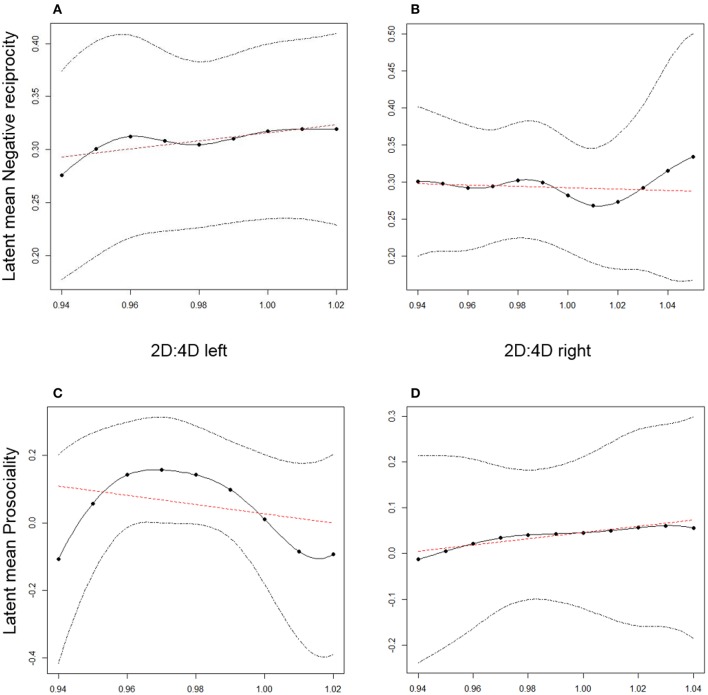
Parameter gradients for latent factor means as estimated by Local Structural Equations to test non-linear associations between 2D:4D and prosociality. In **(A,C)** the x-axis depicts average values of the measured 2D:4D across the two raters at the left hand side. In **(B,D)** the x-axis depicts average values of the measured 2D:4D across the two raters at the right hand side.

Local Structural Equation Models (LSEM, see above) were estimated for negative reciprocity and prosociality along the left vs. right hand 2D:4D measures in four separately fitted one factorial models. 2D:4D left vs. right were considered measured moderator variables for LSEM, with their values resulting by averaging the two available ratings from two different raters. LSEM models were run for the whole sample including females and males. The parameter of interest is the factor mean for negative reciprocity and prosociality as a gradient across the values of 2D:4D for left vs. right hand. Thus, latent factors were scaled by a reference indicator concerning the covariance as well as the mean structure in order to obtain estimates of latent factor means (see for example Little et al., 2007 for details regarding scaling of latent factors). Figure 5 displays the gradients for the latent mean of the negative reciprocity factor (Figure 5A—left hand 2D:4D and negative reciprocity; Figure 5B—right hand 2D:4D and negative reciprocity) and the latent mean of the prosociality factor (Figure 5C—left hand 2D:4D and prosociality; Figure 5D—right hand 2D:4D and prosociality) along with confidence intervals. The gradients suggest an inverted U-shaped relation only for prosociality and left hand 2D:4D. Because the non-linear association is only visible at the left hand, we must treat this finding with caution.

## Discussion

The aim of this study was to investigate the relationship of static markers of testosterone with facets of socio-economic decision-making. Based on the biosocial model of status (Mazur, 1985) we hypothesized static markers indicating higher levels of testosterone to be associated with status defending or assertive behavior. In order to test this hypothesis we had independent raters assess 2D:4D and WHR in a sample of *N* = 175 participants who played the ultimatum game. Respondent behavior in UG captures the tendency to reject unfair offers (negative reciprocity). If unfair UG offers are seen as provocations, then individuals with stronger testosterone exposition may be more prone to reject such offers. Economists argue that negative reciprocity reflects altruistic punishment, since the rejecting individual is sacrificing own resources (Fehr and Gächter, 2002). However, recent studies suggest that self-interest, in terms of status defense plays a substantial role in decisions to reject unfair offers (Yamagishi et al., 2009, 2012; Kaltwasser et al., 2016). We also assessed social preferences by social value orientation (SVO) as an indicator for prosociality and assertiveness via self-report.

We estimated the latent level association of 2D:4D and WHR with negative reciprocity, assertiveness and prosociality in both sexes. To our knowledge, this is the first study combining prenatal and pubertal static indicators within one model of socio-economic decision-making. Results revealed no robust sex-specific association between any of the trait measures and hormonal markers. When collapsing across sex greater WHR was weakly associated with assertiveness (β = 0.20) and the right hand 2D:4D was weakly associated with prosocial behavior (β = 0.21). Furthermore, the measures of 2D:4D and WHR were not related with each other. While the study yielded mainly non-significant results, the findings are interesting and meaningful, as they seem to substantiate the inferences and conclusions offered in several recently published studies and meta-analyses.

In view of the hypothesized relationships, our results are in line with findings of various studies reporting nil correlation of 2D:4D with trait measures such as assertiveness (Hampson et al., 2007; Voracek, 2009), depression (Yeung and Tse, 2017) or indices of socio-economic behavior such as financial risk preferences (Apicella et al., 2008). As presented in Figure 3, only when collapsing across gender the right hand 2D:4D was significantly, albeit weakly associated with prosocial behavior, indicating that individuals with lower prenatal testosterone exposure are somewhat more cooperative. Previous research linking 2D:4D to cooperation behavior suggests that there is no linear relationship between prenatal testosterone exposure and prosociality, but that the relationship is rather U-shaped (non-monotonic) in that subjects with both high and low digit ratios give less than individuals with intermediate digit ratios. However, the existing studies supporting this claim differ in the tested sample regarding gender and the tested criterion regarding hand as well as in the applied socio-economic paradigm, so that a systematic conclusion is impossible. For example, Brañas-Garza et al. (2013) investigated the relationship between cooperation in the dictator game and 2D:4D and found an inverted U-shaped relation for left and right hands in both genders, with a more consistent relationship in men. Sanchez-Pages and Turiegano (2010) only studied the right hand in a male population and report intermediate 2D:4D as being associated with higher cooperation in a Prisoner's Dilemma. The picture gets more complicated as ethnicity also might play a role in that a robust non-monotonic association can only be replicated for Caucasian subjects in the right hand (Galizzi and Nieboer, 2015). In this respect, our study can contribute a valuable piece of evidence to the hypothesized relationship between cooperation and 2D:4D since we tested and compared both genders in both hands in a fairly large Caucasian sample. Our results suggest a small association between right-hand 2D:4D and prosocial behavior in terms of SVO and giving in UG, which neither is modulated by gender nor does it show a non-monotonic relationship for the right hand. However, there seems to be some evidence for an inverted u-shaped relationship between prosociality and left hand's 2D:4D in our sample (see Figure 5C).

Failure to detect significant 2D:4D effects has also been attributed to methodological weaknesses of a study, such as sample structure, its' heterogeneity or size, and also reliability issues related to 2D:4D measurement (e.g., Apicella et al., 2008). These arguments, however, cannot apply to our data having in mind the recruitment procedures and the effective degrees of freedom in this study (see Methods section) as well as the 2D:4D measurement procedure and method employed which followed the findings of previous evaluations of their reliability (Mikac et al., 2016). Moreover, as obvious from the analyses presented, all the study variables including 2D:4D measurements were defined by multiple indicators, that is, on a latent level and hence being free of measurement error.

Less clear empirical evidence is available on the role of facial WHR, with generally modest effect sizes reported where links were detected between WHR and selected target variables, typically referring to aggressive and/or dominant behavior (Geniole et al., 2015; Haselhuhn et al., 2015; Anderl et al., 2016). Comparable to the results we obtained for 2D:4D data, only after collapsing across sex greater WHR in our study appeared to be weakly associated with assertiveness, suggesting that individuals with wider faces tend to express greater status defense. Still, rejection behavior in UG was not related with 2D:4D nor WHR in any of the models. This applies to the tests of both linear and non-linear relationships between the indices of organizational effects of hormones and the behavioral measures examined. Hence, neither hypothesized inverted U-shaped relation of digit ratio with prosociality nor U-shaped with negative reciprocity can be supported by this study.

Zero correlation found between latent 2D:4D and WHR deserves additional comment. This result is not surprising bearing in mind the upheld meaning and the rationale behind each of the two measures. While both are being considered to reflect organizational effects of exposure to sex steroids, they have been linked to different developmental stages–2D:4D being used as a proxy for pre- or perinatal testosterone exposure and WHR as a marker for pubertal hormone exposure. As no substantial correspondence is expected between perinatal and pubertal testosterone levels, the absence of a correlation between the two indicators is plausible (although see Whitehouse et al., 2015). In a similar vein, statistical independence found between 2D:4D and several related sexually dimorphic facial metric measures (Burriss et al., 2007), as well as between each of these putative markers with circulating level of testosterone, has even been suggested as an evidence of their discriminant validity as measures of androgenization in respective time periods (Apicella et al., 2008).

Yet, there is also data advancing that sexually dimorphic features reflected in differing facial growth attributes might originate much earlier than pubertal age and that variation in facial WHR might begin as early as prenatal development (Bird et al., 2016). Whitehouse et al. (2015) showed that adult morphology happened to be more closely related to prenatal testosterone exposure than to adult concentrations, not ruling out, though, possible influences of adolescence testosterone levels. In a comprehensive 20-years follow-up study, these authors provided the direct evidence of a considerable association between prenatal testosterone exposure and human facial structure. Yet, this link was established between prenatal testosterone measured from umbilical cord blood and facial masculinity quantified by an objective algorithm based on multiple Euclidean and geodesic distances on 3D facial photography. Importantly, no relations were detected in the same study between WHR and 2D:4D indices, nor between each of the two static markers with either umbilical cord blood testosterone, adult testosterone level or the derived facial “genderness” score.

It seems that insights from this and other above mentioned studies including our own can at least partly account for the obtained overall modest and practically negligent findings on the relationships between the putative markers of testosterone exposure and behavioral trait measures. The results presented in this study support the position of a number of authors who question the status of either or both the digit ratio and facial WHR as static biomarkers for the assessment of prenatal and pubertal level of testosterone, respectively, or testosterone related traits (Hollier et al., 2015; Hodges-Simeon et al., 2016; Welker et al., 2016; Kramer, 2017; Yeung and Tse, 2017).

Our results propose that previous studies over-estimated the influence of static markers of testosterone on aggression and competition behavior in males. Moreover, when interpreting the role of testosterone in status-related behavior such as socio-economic decision making one should distinguish between static and dynamic markers of testosterone and take into account the situational dependency of the latter (Eisenegger et al., 2010; van Honk et al., 2012). Hence, we suggest that future studies should investigate the behavioral consequences of biological markers as a proxy for hormonal exposure more carefully, essentially relying on multimethod data (e.g., Brañas-Garza et al., in press) and prudently chosen methodological approaches to analyze them, primarily depending on research design and metric quality of the data. Thus, structurally different biological markers of testosterone (static as 2D:4D and dynamic markers measured as circulating blood levels) could potentially be combined with different behavioral indicators of cooperation and analyzed preferably using latent variable modeling approach within a multi-trait-multi-method framework (MTMM; Eid and Diener, 2006).

Last but not least, we would like to emphasize that while it reflects ecologically valid real-world strategic social decision making, behavior in socio-economic games is not as uniform as it is often claimed—a matter that has been discussed recently in the literature (Wilhelm et al., 2017). For example, while economists and psychologists agree that specific socio-economic paradigms such as the dictator game and the Prisoner's Dilemma unequivocally measure common aspects of altruistic or cooperative behavior (Levitt and List, 2007), they consent less on the question whether positive reciprocity (e.g., prosociality) and negative reciprocity (e.g., rejection of unfair offers) reflect two sides of the same coin (Yamagishi et al., 2012; Peysakhovich et al., 2014) as suggested in the theory of altruistic punishment. Furthermore, other aspects of socio-economic decision-making such as risk-taking or uncertainty avoidance should be taken into account in future studies relating facets of socio-economic decision-making to testosterone (Brañas-Garza and Rustichini, 2011).

## Author contributions

LK, Conceptual design, development of socio-economic paradigm, data acquisition, data analysis, writing of manuscript. UM, Conceptual design, data acquisition, data preprocessing, literature research. VB, Conceptual design, data analysis, writing of manuscript. AH, Conceptual design, data analysis (LSEM), writing of manuscript.

### Conflict of interest statement

The authors declare that the research was conducted in the absence of any commercial or financial relationships that could be construed as a potential conflict of interest.
