# Corrigendum: No Robust Association between Static Markers of Testosterone and Facets of Socio-Economic Decision Making

**DOI:** 10.3389/fnbeh.2018.00032

**Published:** 2018-02-26

**Authors:** Laura Kaltwasser, Una Mikac, Vesna Buško, Andrea Hildebrandt

**Affiliations:** ^1^Berlin School of Mind and Brain, Humboldt-Universität zu Berlin, Berlin, Germany; ^2^Faculty of Humanities and Social Sciences, University of Zagreb, Zagreb, Croatia; ^3^Department of Psychology, Ernst-Moritz-Arndt-Universität Greifswald, Greifswald, Germany

**Keywords:** testosterone, 2D:4D, facial width-to-height ratio, economic decision making, social preferences, assertiveness

In the original article, we neglected to include the funder **(German Research Foundation and the Open Access Publication Fund of the University of Greifswald)** to **(Andrea Hildebrandt)**. The authors apologize for this error and state that this does not change the scientific conclusions of the article in any way.

The original article has been updated.

## Conflict of interest statement

The authors declare that the research was conducted in the absence of any commercial or financial relationships that could be construed as a potential conflict of interest.

